# Clinical significance and outcomes of bilateral and unilateral recurrent laryngeal nerve lymph node dissection in esophageal squamous cell carcinoma: A large‐scale retrospective cohort study

**DOI:** 10.1002/cam4.4399

**Published:** 2022-02-17

**Authors:** Shuishen Zhang, Qianwen Liu, Bin Li, Minghan Jia, Xiaoli Cai, Weixiong Yang, Shufen Liao, Zhongkai Wu, Chao Cheng, Jianhua Fu

**Affiliations:** ^1^ Department of Thoracic Surgery The First Affiliated Hospital Sun Yat‐Sen University Guangzhou People’s Republic of China; ^2^ Guangdong Esophageal Cancer Institute Guangzhou People’s Republic of China; ^3^ Department of Thoracic Oncology Sun Yat‐Sen University Cancer Center Guangzhou People’s Republic of China; ^4^ State Key Laboratory of Oncology in South China Collaborative Innovation Center for Cancer Medicine Sun Yat‐Sen University Cancer Center Guangzhou People’s Republic of China; ^5^ Biostatistics Team Clinical Trials Unit The First Affiliated Hospital Sun Yat‐Sen University Guangzhou People’s Republic of China; ^6^ Department of Breast Cancer Guangdong Provincial People's Hospital Cancer Center Guangdong Academy of Medical Sciences Guangzhou Guangdong China; ^7^ Department of Medical Ultrasonics First Affiliated Hospital of Jinan University Guangzhou People’s Republic of China; ^8^ The First Affiliated Hospital of Sun Yat‐Sen University Guangzhou People’s Republic of China; ^9^ Department of Cardiac Surgery The First Affiliated Hospital of Sun Yat‐Sen University Guangzhou People’s Republic of China; ^10^ Key Laboratory on Assisted Circulation Ministry of Health Guangzhou People’s Republic of China

**Keywords:** esophageal squamous cell carcinoma, inverse probability of treatment weighting, morbidity, prognosis, recurrent laryngeal nerve lymph nodes dissection

## Abstract

**Background:**

The survival benefits of recurrent laryngeal nerve lymph node dissection (RLNLD) in esophageal squamous cell carcinoma (ESCC) are still under debate, and the prognostic value of unilateral RLNLD has been rarely studied. Therefore, the aim of the present study was to investigate the clinical significance and outcomes of RLNLD in ESCC in a large‐scale cohort study, to shed light on the outcomes of unilateral RLNLD, and to identify the factors that affect the prognostic outcome of RLNLD.

**Methods:**

We retrospectively reviewed 1153 patients with thoracic ESCC who underwent right thoracotomy with lymphadenectomy. The impact of RLNLD on disease‐free survival (DFS) and overall survival (OS) was estimated using the Kaplan–Meier method and Cox proportional hazard models. Inverse probability of treatment weighting (IPTW) was performed to adjust for differences in baseline variables in pairwise comparisons. Subgroup analysis of survival and postoperative complications was conducted for selective RLNLD.

**Results:**

RLN lymph node (LN) metastasis was independently associated with tumor location and most other LN station metastases. RLNLD was an independent prognostic factor for DFS and OS. Both patients who underwent unilateral and bilateral RLNLD had significantly better DFS and OS than the non‐RLNLD patients. Furthermore, pairwise comparisons with IPTW confirmed these results, and we found that patients who underwent bilateral RLNLD had better survival than those who underwent unilateral RLNLD. However, subgroup analysis showed that there was no survival benefit and higher morbidity after bilateral RLNLD for patients with cancer in the lower thoracic esophagus, and elderly and female patients.

**Conclusion:**

RLN LN metastasis is very frequent in ESCC, and both unilateral and bilateral RLNLD have considerable survival benefits. Selective RLNLD with better survival and lower morbidity was recommend for some defined subgroups.

## Graphical abstract

Unilateral as well as bilateral recurrent laryngeal nerve lymph node dissection (RLNLD) have better long‐term survival than non‐RLNLD. Selective RLNLD with better survival and less morbidity was recommend for some defined subgroups.

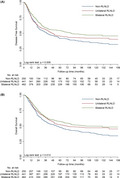

## 1 INTRODUCTION

Esophagectomy is the mainstay of treatment for esophageal cancer patients without systemic metastases. Additionally, lymphadenectomy has been shown to be important for pathological staging and treatment of esophageal cancer.^1^ There exists a trade‐off between the potential survival benefit with more extensive lymphadenectomy and decreased postoperative morbidity with less extensive lymphadenectomy.^2^ Controversy exists regarding the optimal lymphadenectomy and the extent of lymph nodes (LNs) dissection in esophageal cancer.^3^

The metastatic rate of recurrent laryngeal nerve (RLN) LNs is the highest in thoracic esophageal squamous cell carcinoma (ESCC).^4^, ^5^ Recurrent laryngeal nerve lymph node dissection (RLNLD) is beneficial because the clearing of LNs reduces the local recurrence in thoracic ESCC.^3^ However, there is no consensus on its prognostic value in the treatment of esophageal cancer.^6^, ^7^, ^8^, ^9^, ^10^, ^11^, ^12^ Because of the deep anatomical location and poor visibility of RLN LNs in the surgical field, bilateral RLNLD is one of the most technically difficult procedures and requires advanced dissection skills. Additionally, bilateral RLNLD is associated with increased incidence of postoperative morbidities such as vocal cord paresis, respiratory failure, and anastomotic fistula, which lead to poor long‐term quality of life and financial burden.^13^ Consequently, many surgeons are hesitant about performing this procedure, and it is recommended that new surgeons perform bilateral RLNLD after achieving a certain level of competence with right RLNLD.^10^, ^14^ Unilateral (right or left) RLNLD is widely accepted and performed by even inexperienced surgeons in routine clinical practice. However, few studies focus on the prognostic value of RLNLD, especially unilateral RLNLD, on the long‐term survival of patients with ESCC.

Therefore, we have retrospectively reviewed 1153 thoracic ESCC patients who underwent esophagectomy through right thoracotomy with lymphadenectomy. We conducted the comparison among non‐RLNLD, unilateral RLNLD, and bilateral RLNLD patients on survival using inverse probability of treatment weighting (IPTW) and subgroup analysis for selective RLNLD. Our findings shed light on the long‐term clinical significance of RLNLD in ESCC patients.

## 2 MATERIALS AND METHODS

### 2.1 Patients

We identified 2412 consecutive patients with esophageal cancer who underwent surgical resection at Sun Yat‐Sen University Cancer Center between December 2000 and December 2008. Patients with thoracic ESCC who underwent esophagectomy via right thoracotomy with lymphadenectomy were included. Patients were excluded based on the following criteria: a history of other cancers, cancer of the cervical or esophagogastric junction, prior neoadjuvant therapy, death in the perioperative period, no radical resection or LN dissection, left‐sided thoracotomy or the transhiatal approach, and lack of follow‐up data (Figure S1). Finally, 1153 patients who met the criteria were enrolled in our study. We collected data on patient characteristics and postoperative complications from a retrospective review of their medical records. All patients were clinically staged according to the findings of chest and abdomen computed tomography (CT) scan or positron emission tomography/CT, endoscopic ultrasound examination, and bronchoscopy. Pathologic stage was determined according to the seventh edition of the AJCC staging system.^15^ The study was approved by the Ethics Committee of Sun Yat‐Sen University Cancer Center. All patients provided their written informed consent.

### 2.2 Surgical procedure

The surgical procedure was performed as previously described in our study.^16^ A right transthoracic esophagectomy was performed with lymphadenectomy. Lymphadenectomy, including standard lymphadenectomy, extended lymphadenectomy, and total lymphadenectomy, was performed according to the guidelines of the Consensus Conference of the International Society for Diseases of the Esophagus in 1994.^17^ RLNLD was performed by detecting and dissecting the entire RLN. The right RLN dissection extended from the lower edge of the brachiocephalic artery to the lower edge of the thyroid, and the left RLN extended from the upper edge of the aortic arch to the lower edge of the thyroid.

### 2.3 Follow‐up and outcomes

Standardized follow‐up was performed as described in our previous study.^16^ Follow‐up was continued until December 2019. Overall survival (OS) and disease‐free survival (DFS) were the primary endpoints, as described in our previous study.^16^

### 2.4 Definition of postoperative complications

The definitions of postoperative complications, including respiratory disease complications (such as pneumonia and respiratory failure), anastomotic leakage, chylous leakage, wound infection, vocal cord paresis, and cardiovascular diseases, are described in detail in our previous study.^16^ All complications that occurred from surgery to discharge from hospital were well documented.

### 2.5 Statistical analysis

Categorical variables were expressed as a number (percentile), and differences in categorical variables were analyzed using the chi‐squared test or the Fisher's exact test. Logistic regression analysis was used to analyze the risk factors for RLN LN metastasis. Survival curves were drawn by the Kaplan–Meier method and analyzed with the log‐rank test. Univariable and multivariable Cox regression models were used to analyze the impact of the observed variables on prognosis. The proportional hazards assumption was tested with the Schoenfeld residuals method, and no significant violation was found for each of the covariates as well as the global test. Pairwise comparisons were conducted between the non‐RLNLD, unilateral RLNLD, and bilateral RLNLD patients, and IPTW was used to adjust for differences in baseline variables between two groups.^18^

All statistical analyses were performed using the Stata/MP 14.0 software. All tests were two‐sided. *p* < 0.05 indicated statistically significant differences, while *p* < 0.017 indicated statistically significant differences for pairwise comparisons.

## 3 RESULTS

### 3.1 Patient characteristics

The selected 1153 ESCC patients were divided into three groups based on RLNLD: 250 (21.7%) patients who did not undergo RLNLD (non‐RLNLD), 441 (38.2%) patients who underwent right or left RLNLD (unilateral RLNLD), and 462 (40.1%) patients who underwent right and left RLNLD (bilateral RLNLD). Significant differences were found among the three groups with regard to gender (*p* = 0.016), pN stage (*p* < 0.001), tumor location (*p* = 0.004), and pTNM stage (*p* = 0.001) (Table 1).

**TABLE 1:** The clinical and pathological characteristics at baseline

Characteristic	Overall (*n* = 1153)	Non RLNLD (*n* = 250)	Unilateral RLNLD (*n* = 441)	Bilateral RLNLD (*n* = 462)	*p* value
Age					0.225
≤60 years	663 (57.5)	154 (61.6)	242 (54.9)	267 (57.8)	
>60 years	490 (42.5)	96 (38.4)	199 (45.1)	195 (42.2)	
Gender					**0.016**
Females	270 (23.5)	72 (28.8)	108 (24.5)	90 (19.5)	
Males	883 (76.5)	178 (71.2)	333 (75.5)	372 (80.5)	
Smoking					0.346
Never	440 (38.2)	102 (40.8)	173 (39.2)	165 (35.7)	
Ever (former + current)	713 (61.8)	148 (59.2)	268 (60.8)	297 (64.3)	
Alcohol					0.186
Never	772 (67.0)	173 (69.2)	304 (68.9)	295 (63.9)	
Ever (former + current)	381 (33.0)	77 (30.8)	137 (31.1)	167 (36.1)	
pT stage					0.534
T1–2	366 (31.7)	82 (32.8)	146 (33.1)	138 (29.9)	
T3–4	787 (68.3)	168 (67.2)	295 (66.9)	324 (70.1)	
pN stage					**<0.001**
N0	562 (48.7)	159 (63.6)	210 (47.6)	193 (41.8)	
N1–3	591 (51.3)	91 (36.4)	231 (52.4)	269 (58.2)	
Differentiation					0.539
G1–2	819 (71.0)	182 (72.8)	317 (71.9)	320 (69.3)	
G3	334 (29.0)	68 (27.2)	124 (28.1)	142 (30.7)	
Tumor location					**0.004**
Upper	232 (20.1)	70 (28.0)	85 (19.3)	77 (16.7)	
Middle	787 (68.3)	148 (59.2)	309 (70.1)	330 (71.4)	
Lower	134 (11.6)	32 (12.8)	47 (10.7)	55 (11.9)	
TNM stage					**0.001**
I	119 (10.3)	28 (11.2)	49 (11.1)	42 (9.1)	
II	527 (45.7)	139 (55.6)	196 (44.4)	192 (41.6)	
III	507 (44.0)	83 (33.2)	196 (44.4)	228 (49.4)	
Adjuvant therapy					0.430
No	926 (80.3)	208 (83.2)	351 (79.6)	367 (79.4)	
Yes	227 (19.7)	42 (16.8)	90 (20.4)	95 (20.6)	
Postoperative complication	453 (39.3)	87 (34.8)	154 (34.9)	212 (45.9)	**0.001**
Respiratory	208 (18.0)	28 (11.2)	70 (15.9)	110 (51.9)	**<0.001**
Anastomotic leakage	212 (18.4)	50 (20.0)	68 (15.4)	94 (20.3)	0.112
Chylous leakage	21 (1.8)	2 (0.8)	13 (2.9)	6 (1.3)	0.071
Wound infection	36 (3.1)	15 (6.0)	10 (2.3)	11 (2.4)	**0.013**
Vocal cord paresis	55 (4.7)	6 (2.4)	17 (3.9)	32 (6.9)	**0.013**
Cardiovascular	49 (4.2)	12 (4.8)	15 (3.4)	22 (4.7)	0.532

Note

G: grade; Adjuvant therapy: 36 patients for chemoradiotherapy, 141 patients for chemotherapy, and 50 patients for radiotherapy.

Bold values are statistically significant (*p* < 0.05).

Abbreviation: RLNLD, recurrent laryngeal nerve lymph node dissection; TNM, tumor node metastasis.

The incidence of postoperative complications after bilateral RLNLD was 45.9% across the three groups (*p* = 0.001). The incidence rates of respiratory diseases and vocal cord paresis after bilateral RLNLD were 51.9% and 6.9%, respectively, which are dramatically higher than the incidence rates after unilateral RLNLD and non‐RLNLD (*p* < 0.05). The incidence of postoperative complications was similar in the unilateral RLNLD and non‐RLNLD groups. However, no significant difference was observed in the incidence of anastomotic leakage, chylous leakage, or cardiovascular diseases among the three groups.

### 3.2 Patient prognosis

**FIGURE 1:**
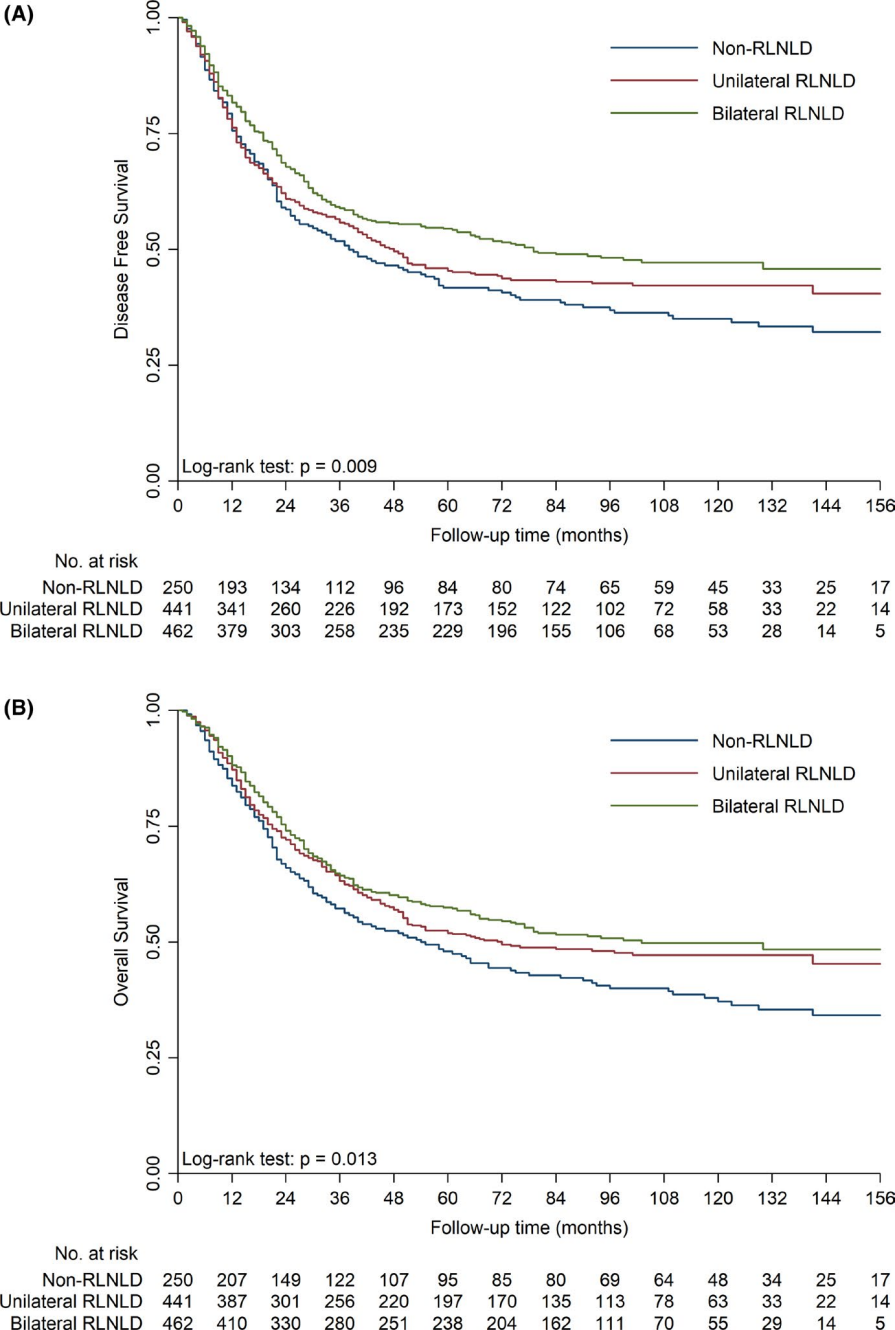
Effect of RLNLD on survival outcomes. The Kaplan–Meier curves show a significant difference in DFS (A) and OS (B) among unilateral RLNLD, bilateral RLNLD, and non‐RLNLD patients. DFS, disease‐free survival; OS, overall survival; RLNLD, recurrent laryngeal nerve lymph node dissection

The median follow‐up time was 48 months. The Kaplan–Meier curves showed that there was a significant difference in DFS (*p* = 0.009, Figure 1A) and OS (*p* = 0.013, Figure 1B) among the three groups. Both unilateral and bilateral RLNLD were associated with significantly better DFS and OS than non‐RLNLD. Moreover, multivariate analysis found that RLNLD was an independent prognostic factor for DFS and OS in patients with ESCC, along with alcohol consumption, pT stage, pN stage, tumor location, and postoperative complications (Table 2). Compared to the non‐RLNLD patients, those who underwent unilateral RLNLD had better DFS (*p* = 0.031) and OS (*p* = 0.002), as did patients who underwent bilateral RLNLD, who also had favorable DFS and OS (*p* < 0.001).

**TABLE 2:** Univariate and multivariate survival analyses for disease‐free survival and overall survival in patients with esophageal squamous cell carcinoma (ESCC)

Prognostic factor	Univariate survival analysis				Multivariate survival analysis			
	DFS		OS		DFS		OS	
	HR (95% CI)	*p* value	HR (95% CI)	*p* value	HR (95% CI)	*p* value	HR (95% CI)	*p* value
Age	1.09 (0.93, 1.28)	0.286	1.21 (1.03, 1.43)	**0.023**	1.15 (0.98, 1.35)	0.093	1.29 (1.09, 1.53)	**0.003**
Gender	0.68 (0.56, 0.83)	<0.001	0.69 (0.56, 0.84)	**<0.001**	0.79 (0.60, 1.02)	0.073	0.82 (0.63, 1.09)	0.171
Smoking	1.33 (1.12, 1.57)	0.001	1.36 (1.14, 1.62)	**0.001**	1.03 (0.82, 1.29)	0.799	1.05 (0.83, 1.33)	0.673
Alcohol	1.43 (1.22, 1.69)	<0.001	1.52 (1.28, 1.79)	**<0.001**	1.27 (1.06, 1.52)	**0.011**	1.39 (1.15, 1.68)	**0.001**
pT stage	1.92 (1.60, 2.31)	<0.001	1.92 (1.59, 2.34)	**<0.001**	1.61 (1.33, 1.95)	**<0.001**	1.64 (1.34, 2.00)	**<0.001**
pN stage	2.31 (1.96, 2.73)	<0.001	2.25 (1.89, 2.67)	**<0.001**	2.21 (1.85, 2.63)	**<0.001**	2.20 (1.83, 2.64)	**<0.001**
Differentiation	1.21 (1.02, 1.43)	0.030	1.15 (0.96, 1.37)	0.134	1.10 (0.93, 1.31)	0.280		
Tumor location								
Upper	1		1		1		1	
Middle	0.76 (0.63, 0.92)	0.004	0.74 (0.61, 0.89)	**0.002**	0.71 (0.58, 0.86)	**<0.001**	0.69 (0.57, 0.84)	**<0.001**
Lower	0.57 (0.42, 0.77)	<0.001	0.54 (0.39, 0.74)	**<0.001**	0.48 (0.35, 0.66)	**<0.001**	0.46 (0.33, 0.65)	**<0.001**
Adjuvant therapy	1.34 (1.11, 1.62)	0.002	1.17 (0.96, 1.43)	0.126	0.99 (0.81, 1.20)	0.887	0.88 (0.71, 1.09)	0.249
Postoperative Complication	1.20 (1.02, 1.41)	0.024	1.26 (1.06, 1.48)	**0.007**	1.20 (1.02, 1.41)	**0.025**	1.24 (1.05, 1.47)	**0.011**
RLNLD								
Non	1		1		1		1	
Unilateral	0.88 (0.71, 1.07)	0.199	0.80 (0.65, 0.99)	**0.042**	0.80 (0.65, 0.98)	**0.031**	0.71 (0.57, 0.88)	**0.002**
Bilateral	0.73 (0.60, 0.90)	0.003	0.73 (0.59, 0.90)	**0.004**	0.60 (0.48, 0.74)	**<0.001**	0.59 (0.48, 0.74)	**<0.001**

Note

Bold values are statistically significant (*p* < 0.05).

Abbreviations: 95% CI, 95% confidence interval; DFS, disease‐free survival; HR, hazard ratio; OS, overall survival; RLNLD, recurrent laryngeal nerve lymph node dissection.

### 3.3 Pairwise comparisons with IPTW

**FIGURE 2:**
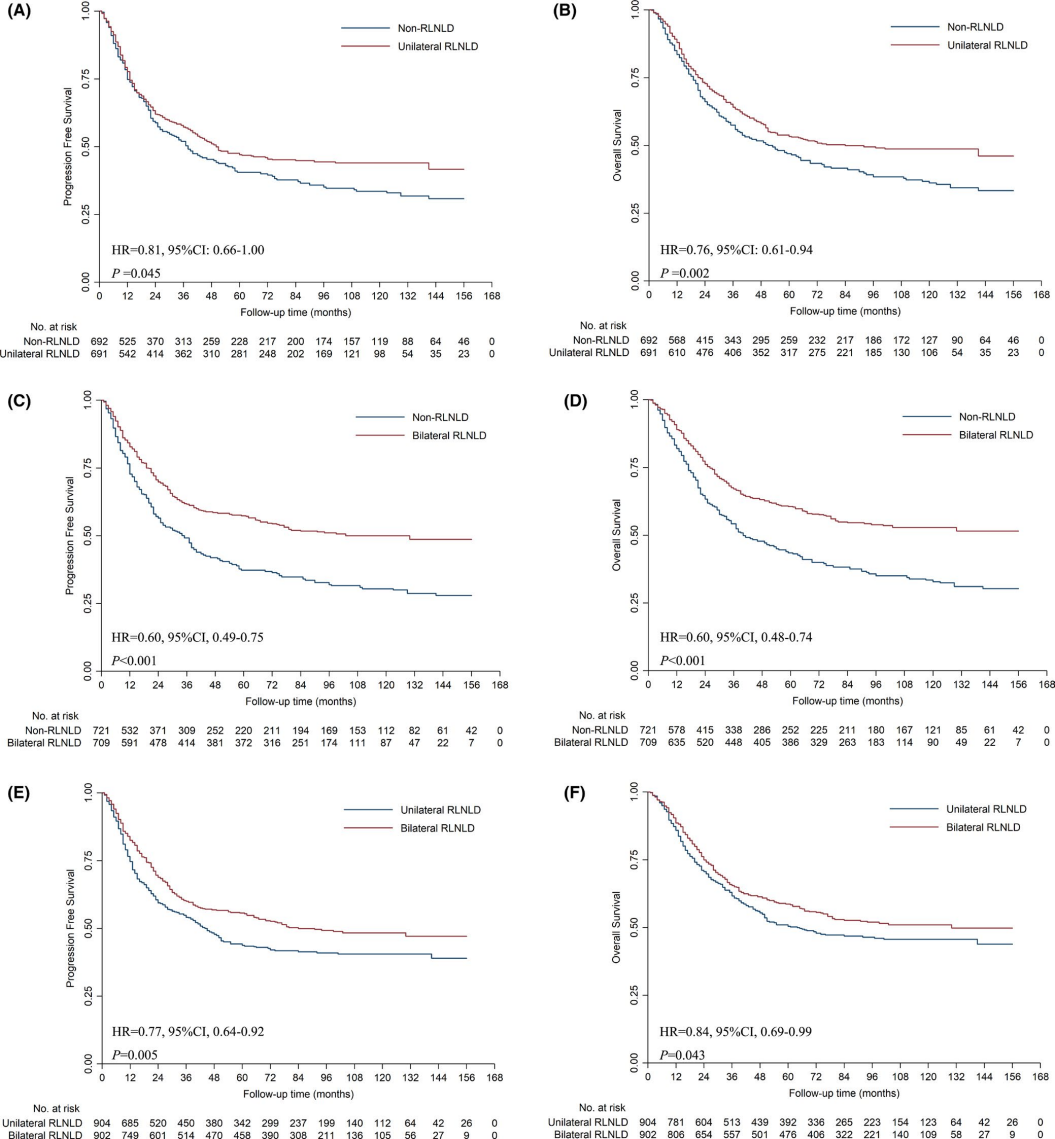
Pairwise comparisons of survival after RLNLD. The Kaplan–Meier curves showed that patients who underwent unilateral RLNLD had better DFS (A) and OS (B) than those who did not undergo RLNLD, and patients who underwent bilateral RLNLD had better DFS (C) and OS (D) than those who did not undergo RLNLD. Additionally, patients who underwent bilateral RLNLD had better DFS (E) and OS (F) than those who underwent unilateral RLNLD. DFS, disease‐free survival; OS, overall survival; RLNLD, recurrent laryngeal nerve lymph node dissection

Differences in baseline characteristics between the three groups were adjusted with IPTW (Tables S1–S3). Patients who underwent unilateral RLNLD had a 19% and 24% lower risk of disease progression (*p* = 0.045, Figure 2A) and death (*p* = 0.002, Figure 2B) than non‐RLNLD patients, and patients who underwent bilateral RLNLD had a 40% and 40% lower risk of disease progression (*p* < 0.001, Figure 2C) and death (*p* < 0.001, Figure 2D) than non‐RLNLD patients. Moreover, patients who underwent bilateral RLNLD had a 23% and 16% lower risk of disease progression (*p* = 0.005, Figure 2E) and death (*p* = 0.043, Figure 2F) than unilateral RLNLD patients.

### 3.4 Subgroup analysis for selective RLNLD

**FIGURE 3:**
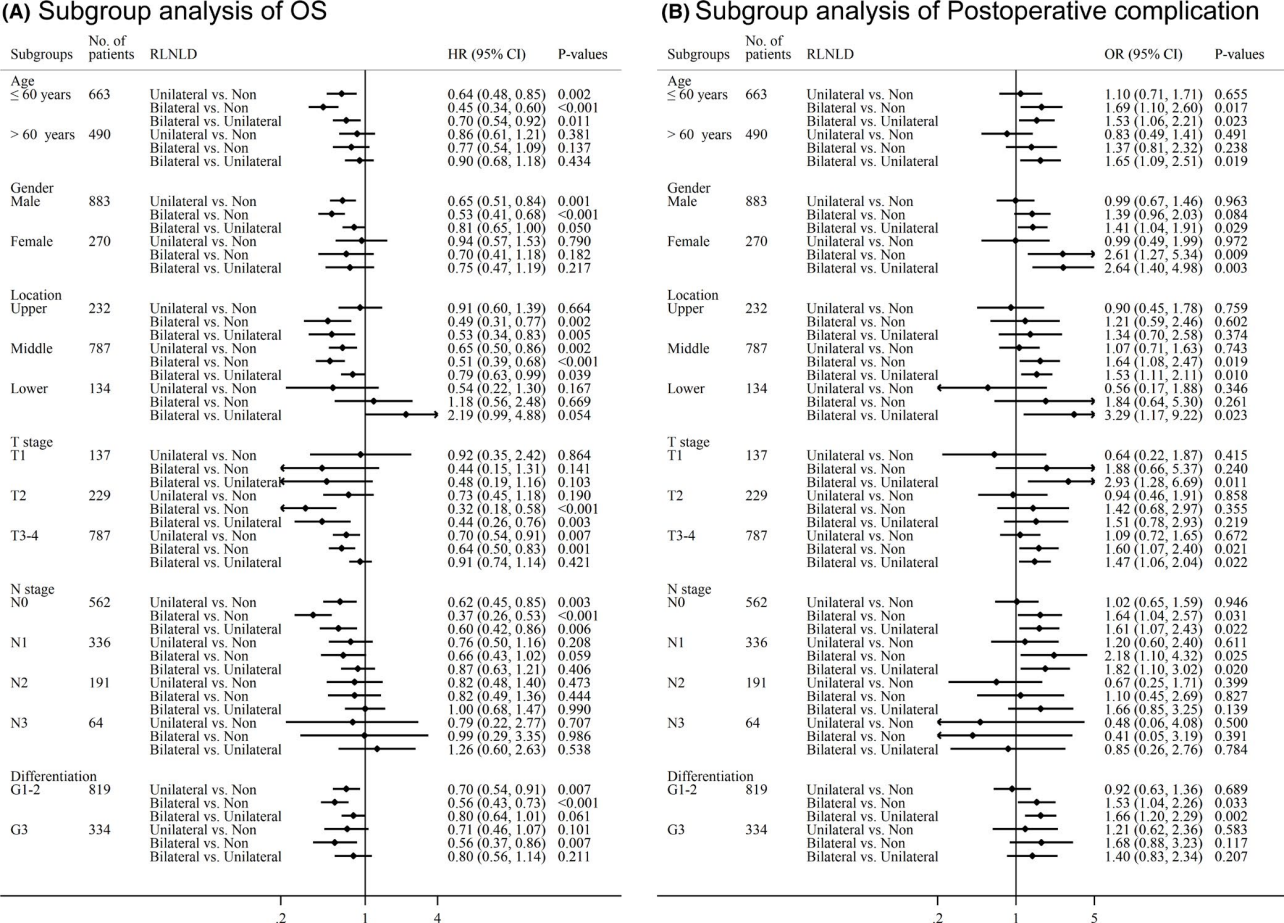
Subgroup analysis of survival after RLNLD. OS (A) and postoperative complications (B) were associated with bilateral RLNLD, unilateral RLNLD, and no RLNLD for various subgroups of patients based on age, gender, pathological stage, degree of differentiation, tumor location, and lymph node involvement. OS, overall survival; RLNLD, recurrent laryngeal nerve lymph node dissection

We conducted subgroup analysis of survival and postoperative complications to determine which factors are associated with better survival and lower morbidity after bilateral, unilateral, and no RLNLD (Figure 3; Figure S2). We found that young age (≤60 years), male sex, cancer in the middle thoracic esophagus, no LN involvement (pN = 0), and a high degree of differentiation (G1–2) were associated with favorable DFS and OS after both unilateral and bilateral RLNLD compared with non‐RLNLD (*p* < 0.05). However, bilateral RLNLD was associated with more postoperative complications, while unilateral RLNLD was similar to non‐RLNLD in this regard. For patients with pathological T2 stage, cancer in the upper thoracic esophagus, and poorly differentiated cancer (G3), bilateral RLNLD resulted in significantly longer DFS and OS than non‐RLNLD, while similar survival rates were observed with unilateral RLNLD and non‐RLNLD. No significant difference in postoperative complications was noted among the three groups. For patients with pathological T3–4 stage, bilateral RLNLD was associated with longer DFS and OS than non‐RLNLD, but bilateral and unilateral RLNLD had similar OS. Additionally, unilateral RLNLD had longer OS than non‐RLNLD. Postoperative complications were more common after bilateral RLNLD than after unilateral RLNLD and non‐RLNLD. Older age, female sex, and cancer in the lower thoracic esophagus were not associated with significant differences in DFS or OS among the three groups. However, in this group of patients, bilateral RLNLD was associated with more postoperative complications than unilateral RLNLD.

### 3.5 Risk factor analysis for RLN LN metastasis

Of the 903 patients who underwent RLNLD, the rate of RLN LN metastasis was higher than that at other LN stations (25.3% in the right and 21.1% in the left, Figures S3 and S4). As shown in Table 3, univariate analysis showed that RLN LN metastasis was positively associated with advanced pT stage and pN stage, poor differentiation, and cancer in the upper and middle thoracic esophagus (*p* < 0.05). Multivariate logistic analysis revealed that tumor location (upper: odds ratio [OR] = 5.04, 95% confidence interval [CI] = 2.59–9.81, *p* < 0.001; middle: OR = 2.82, 95% CI = 1.55–5.13, *p* = 0.001), but not pathological T stage or degree of differentiation, was an independent predictive factor of RLN LN metastasis. Additionally, RLN LN metastasis was independently associated with upper thoracic paraesophageal LN metastasis, subcarinal LN metastasis, right main bronchus LN metastasis, paracardial LN metastasis, and left gastric LN metastasis (*p* < 0.05).

**TABLE 3:** Univariate and multivariate logistic analyses of correlation between clinicopathological factors and RLN LNs metastasis

Characteristic	Univariate analysis			Multivariate analysis	
	RLN LNs negative (*n* = 619)	RLN LNs positive (*n* = 284)	*p* value	OR (95% CI)	*p* value
Age			0.828		
≤60 years	347 (56.1)	162 (57.0)			
>60 years	272 (43.9)	122 (43.0)			
Gender			0.604		
Females	480 (77.5)	225 (79.2)			
Males	139 (22.5)	59 (20.8)			
Smoking			0.882		
Never	233 (37.6)	105 (37.0)			
Ever (former + current)	386 (62.4)	179 (63.0)			
Alcohol			0.448		
Never	416 (67.2)	183 (64.4)			
Ever (former + current)	203 (32.8)	101 (35.6)			
pT stage			**0.020**		
T1–2	210 (33.9)	74 (26.1)		1	
T3–4	409 (66.1)	210 (73.9)		1.12 (0.80, 1.58)	0.497
pN stage			**<0.001**		
N0	403 (65.1)	0 (0.0)			
N1–3	216 (34.9)	284 (100.0)			
Differentiation			**0.018**		
G1–2	452 (73.0)	185 (65.1)		1	
G3	167 (27.0)	99 (34.9)		1.33 (0.96, 1.84)	0.084
Tumor location			**<0.001**		
Upper	95 (15.3)	67 (23.6)		5.04 (2.59,9.81)	**<0.001**
Middle	438 (70.8)	201 (70.8)		2.82 (1.55,5.13)	**0.001**
Lower	86 (13.9)	16 (5.6)		1	
Upper thoracic paraesophageal LNs metastasis	25 (4.0)	28 (9.9)	**0.001**	2.02 (1.11, 3.69)	**0.022**
Pretracheal LNs metastasis	1 (0.2)	5 (1.8)	**0.013**	8.59 (0.96, 76.92)	0.055
Right tracheobronchial LNs metastasis	6 (1.0)	6 (2.1)	0.209	3.93 (1.34, 11.51)	**0.013**
Subcarinal LNs metastasis	39 (6.3)	52 (18.3)	**<0.001**	2.26 (1.39, 3.68)	**0.001**
Middle thoracic paraesophageal LNs metastasis	59 (9.5)	59 (20.8)	**<0.001**	1.54 (0.98, 2.41)	0.061
Left main bronchus LNs metastasis	9 (1.5)	13 (4.6)	**0.009**	1.70 (0.66, 4.40)	0.273
Right main bronchus LNs metastasis	6 (1.0)	12 (4.2)	**0.003**		
Lower thoracic paraesophageal LNs metastasis	40 (6.5)	27 (9.5)	0.132		
Supradiaphragmatic LNs metastasis	5 (0.8)	2 (0.7)	1.000		
Pulmonary ligament LNs metastasis	2 (0.3)	2 (0.7)	0.594		
Paracardial LNs metastasis	46 (7.4)	48 (16.9)	**<0.001**	1.82 (1.11, 2.96)	**0.017**
Lesser curvature nodes metastasis	22 (3.6)	13 (4.6)	0.462		
Left gastric nodes metastasis	63 (10.2)	53 (18.7)	**0.001**	1.61 (1.01, 2.56)	**0.043**
Celiac nodes metastasis	1 (0.2)	2 (0.7)	0.234		

Note

G: grade.

Bold values are statistically significant (*p* < 0.05).

95% CI, 95% confidence interval; LNs, lymph nodes; OR, odds ratio; RLN, recurrent laryngeal nerve.

## 4 DISCUSSION

In the present study, we have conducted detailed analyses of the complications and outcomes of unilateral and bilateral RLNLD for ESCC in order to shed light on how this procedure affects the long‐term prognosis of this cancer. Additionally, we have examined the factors that are associated with the outcomes of these two procedures in order to identify the indications and contradictions in various subgroups of patients. We believe that the findings will be useful for the selection of ESCC patients for bilateral and unilateral RLNLD.

Our current study indicated that unilateral and bilateral RLNLD had better DFS and OS than non‐RLNLD. These findings were confirmed by adjusting for differences in baseline variables and eliminating a selection bias with pairwise comparisons using the IPTW method. In contrast, Fujita et al. reported that there was no significant difference in long‐term survival among bilateral RLNLD, unilateral RLNLD, and non‐RLNLD patients.^7^ Despite this, our results are consistent with those of previous studies which found that bilateral RLNLD resulted in better survival than non‐bilateral RLNLD.^9^, ^10^ However, the previous studies did not investigate the prognostic role of unilateral RLNLD. In this study, we found that patients who underwent unilateral RLNLD had longer survival than those who did not undergo RLNLD, but had worse survival than patients who underwent bilateral RLNLD. To the best of our knowledge, this is the first large‐scale cohort study to demonstrate that both bilateral RLNLD and unilateral RLNLD have convincing long‐term survival benefits in ESCC.

The survival benefits of RLNLD could be associated with the removal of localized LN metastasis and undetected micrometastases. In this study, we observed that among the regional LNs, RLN LNs, especially right RLN LNs, were the most common metastasis sites. In accordance with our findings, high metastasis rates of the RLN LNs as compared to other LN stations have been reported in other studies, too, including a higher rate of metastasis in the right than in the left RLN LNs.^4^ ^,^ ^19^ ^,^ ^20^ Additionally, one study found that RLN LN metastasis was significantly associated with poorer OS and DFS in esophageal cancer.^21^ Therefore, the clearing of RLN LNs may majorly contribute to reducing the local recurrence in patients with thoracic ESCC. Another benefit of RLNLD is that correct pathological stage information can be obtained from the metastatic RLN LNs that are largely harvested. This is confirmed by the observation that patients with higher pN stage (1–3) and advanced pathological stage who underwent RLNLD had better survival than those who did not undergo RLNLD. In addition, in the current study, there was no significant difference in adjuvant therapy between the RLNLD and non‐RLNLD patients. This indicates that the effect of RLNLD on prognosis was not associated with adjuvant therapy. Our previous clinical trial and many studies demonstrated that neoadjuvant chemoradiotherapy prolongs survival in patients with locally advanced esophageal cancer.^22^, ^23^ Previous study reported that bilateral RLNLD can be safely performed in ESCC patients who had undergone neoadjuvant chemoradiotherapy, ultimately resulting in an improved local control. Nevertheless, bilateral RLNLD did not benefit on the overall recurrence rate or survival when compared with non‐bilateral RLNLD.^24^ Neoadjuvant therapy was not the standard treatment for esophageal cancer in our center between December 2000 and December 2008, which leaded to only 176 patients received neoadjuvant therapy (52 patients for chemoradiotherapy, 73 patients for radiotherapy, and 51 patients for chemotherapy) in our cohort. What is worse, the regimen of chemoradiotherapy or chemotherapy varied in different patients. Therefore, it was difficult to elucidate the role of RLNLD on patients who underwent neoadjuvant therapy in our current study. More importantly, preoperative treatment might have confounded the outcomes and hindered statistical analysis in our study. Thus, we excluded patients who received neoadjuvant therapy in order to more focus on examining differences in surgical techniques in operable ESCC as previous studies did.^9^, ^12^

Our findings indicate that bilateral RLNLD caused more postoperative complications, such as respiratory diseases and vocal cord paresis, while unilateral RLNLD was safer than non‐RLNLD. This result is similar to that published by a previous study by Taniyama et al. which reported that patients who underwent bilateral RLNLD had more frequent RLN palsy than those who did not underwent bilateral RLNLD (41% vs. 28%).^10^ Lymphadenectomy along the bilateral RLN frequently results in RLN injury, which leads to serious morbidity, poor quality of life, and financial burden. Bilateral RLNLD is one of the most technically difficult procedures and requires advanced dissection skills. In fact, unilateral RLNLD is widely accepted and performed routinely by new surgeons to minimize postoperative mortality and morbidity during the learning curve.^10^, ^14^ Given the considerable postoperative morbidity associated with bilateral RLNLD, it is important to find a method to determine the optimal RLNLD procedure that could have favorable short‐ and long‐term outcomes for specific subgroups of patients in clinical practice. In fact, Yu et al. reported that RLNLD can be omitted in low‐risk patients with early stage ESCC, as it is associated with significant complications without sufficient survival benefits.^12^ Thus, it is important to explore the indications for RLNLD in order to identify which subgroup of patients can benefit from the procedure.

Few studies have explored the subgroups with indications for RLNLD. In our study, we investigated the risk factors of RLN LN metastasis, as these are regarded as indicators of the extent of lymphadenectomy and unfavorable prognosis of thoracic ESCC.^25^, ^26^, ^27^ Multivariate logistic analysis revealed that upper and middle thoracic esophagus cancer was independently associated with RLN LN metastasis when compared with lower thoracic cancer. Therefore, tumor location should be considered as the most important indicator for RLNLD. This finding is supported by several other studies in the literature which also show that the metastasis of RLN LNs is closely associated with the location of the tumor.^12^, ^19^, ^20^ Accordingly, our subgroup analysis indicated that bilateral RLNLD is indispensable for patients with upper ESCC because it leads to favorable survival without causing more postoperative complications than unilateral or no RLNLD. Furthermore, both bilateral RLNLD and unilateral RLNLD seem to have survival benefits in the case of middle thoracic esophagus cancer. However, bilateral RLNLD should be performed with caution in patients with ESCC in the lower thoracic esophagus as it does not show sufficient survival benefits but is associated with more postoperative complications. In contrast to these findings, Fujita et al. reported that there was no significant difference in long‐term survival among bilateral RLNLD, unilateral RLNLD, and non‐RLNLD patients, irrespective of the location of the tumor.^7^ However, our findings are supported by a previous study which also showed that bilateral RLNLD resulted in a better 5‐year survival rate than non‐bilateral RLNLD in patients with upper and middle thoracic esophagus cancer, whereas patients with lower thoracic esophagus cancer did not benefit from bilateral RLNLD.^10^ Given these inconsistencies, the extent to which tumor location affects the outcome of RLNLD needs to be further explored in future studies on larger cohorts.

In the present study, we also found that the pT stage and degree of differentiation were associated with an increased risk of RLN LN metastasis according to univariate logistic analysis. Other published studies on the risk factors for RLN LN metastasis in ESCC have also shown that the degree of differentiation of tumor tissue is a major risk factor.^20^, ^28^, ^29^ The subgroup analysis in our study shows that bilateral RLNLD is beneficial in patients with pathological stage T2 and poorly differentiated cancer. In accordance with our findings, Taniyama et al. reported that patients in pathological stage T3 benefited from bilateral RLNLD, whereas patients in pathological stage T0–1 and T4 did not benefit from bilateral RLNLD when compared with non‐bilateral RLNLD.^10^ Additionally, Wang and Liu have reported that left para‐RLNLD has better efficacy in stage T2 and T3 patients but is limited in stage T1 patients.^11^ With regard to all the examined risk factors, young age, male sex, pathological stage T3–4, lack of LN involvement, and a high degree of differentiation are associated with survival benefits after both unilateral and bilateral RLNLD. However, bilateral RLNLD is not appropriate procedure for patients with elder age, and female. The indications for RLNLD in patients with pathological stage T1 and LN metastasis are unclear and need to be examined further in the future.

Overall, the findings of our subgroup analysis show that bilateral RLNLD should be considered as a routine lymphadenectomy procedure for cancer of the upper thoracic esophagus, pathological stage T2 cancer, and poorly differentiated cancer. Moreover, considering the higher rate of postoperative complications after bilateral RLNLD and lower morbidity rates after unilateral RLNLD, the latter might be the better option for inexperienced surgeons to minimize complications when the following factors are present: cancer of the middle thoracic esophagus, young age, male sex, pathological stage T3–4, no involvement of the mediastinal LN, and a high degree of differentiation. However, bilateral RLNLD may not have survival benefits and be associated with higher morbidity rates in patients who have cancer of the lower thoracic esophagus and in elderly and female patients, especially if accurate preoperative assessment indicates that the RLN LNs are not involved. In the future, a multicenter randomized clinical trial study should be conducted to understand the clinical significance of RLNLD, especially unilateral RLNLD for ESCC.

There are several limitations in our study. First, although IPTW was used to adjust for variables that may influence the outcomes, our study may still have a selection bias on account of its retrospective design. Second, the value of RLNLD in reducing local recurrence could not be clearly determined due to insufficient information on regional LN recurrence in each patient. Third, the prognostic value and postoperative complications of RLNLD for patients undergoing neoadjuvant therapy need to be studied because patients who underwent neoadjuvant therapy were excluded. We will further focus on this issue by analysis of our clinical trial (NEOCRTEC5010) in next study.^22^ Fourth, the influence of RLNLD on quality of life was not investigated due to the lack of data on quality of life in our study.

## 5 CONCLUSIONS

In conclusion, RLN LN metastasis is not rare in patients with ESCC, and both bilateral and unilateral RLNLD can improve the long‐term survival. However, bilateral RLNLD is associated with more postoperative complications, and unilateral RLNLD is safer. Certain subgroups of patients, for example, younger patients and patients with a higher degree of differentiation, are more eligible than others for bilateral RLNLD and show better survival and less morbidity, while certain other subgroups have contraindications for bilateral RLNLD and may benefit better from unilateral RLNLD. Further prospective studies in larger cohorts of patients are necessary to confirm these results.

## ETHICS STATEMENT

The study was approved by the Ethics Committee of Sun Yat‐sen University Cancer Center. All patients provided their written informed consent.

## Supporting information

Fig S1

Fig S2

Fig S3

Fig S4

Table S1‐S3

## Data Availability

The datasets generated during and analyzed during the current study are available from the corresponding author on reasonable request.
