# Supplementary figures and images for: Clinical significance and outcomes of bilateral and unilateral recurrent laryngeal nerve lymph node dissection in esophageal squamous cell carcinoma: A large‐scale retrospective cohort study
Source: Cancer Med. 2022 Feb 17;11(7):1617–29. doi: 10.1002/cam4.4399 (PMC8986140; doi:10.1002/cam4.4399)

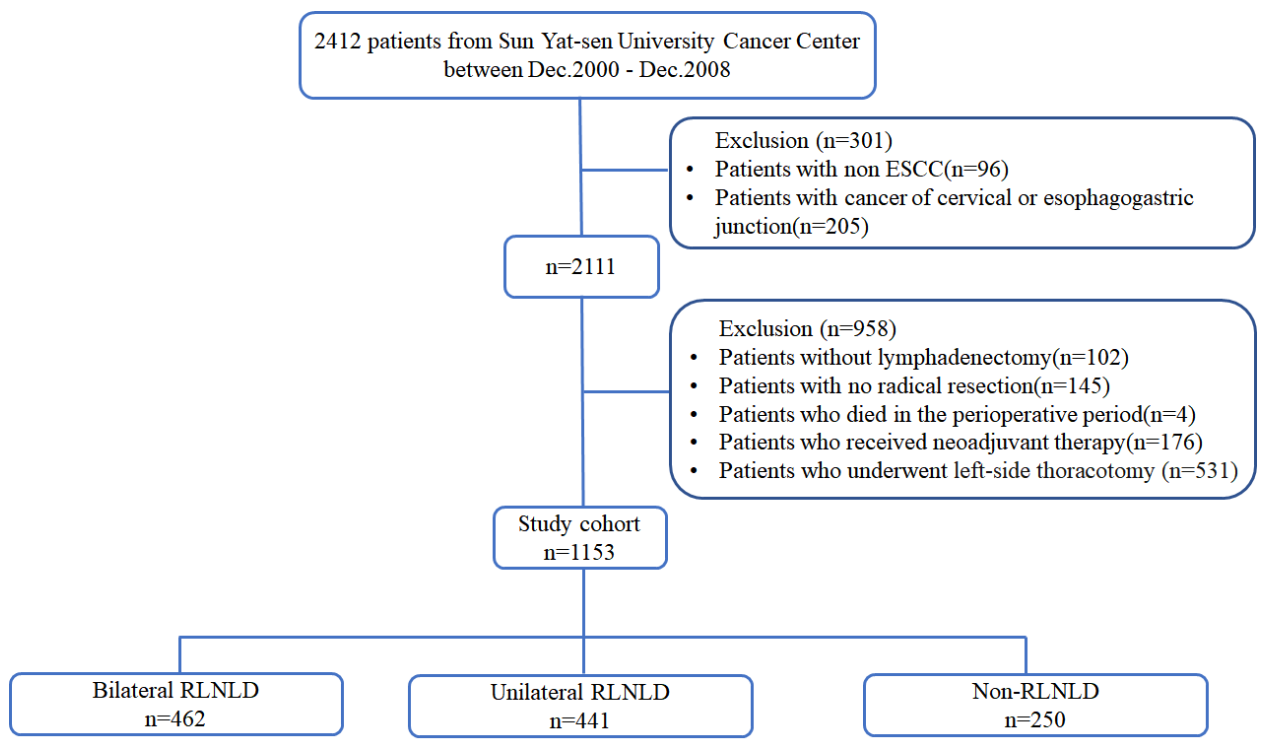

Supplement: Supplementary file 1 — Fig S1 [file CAM4-11-1617-s003.tiff]

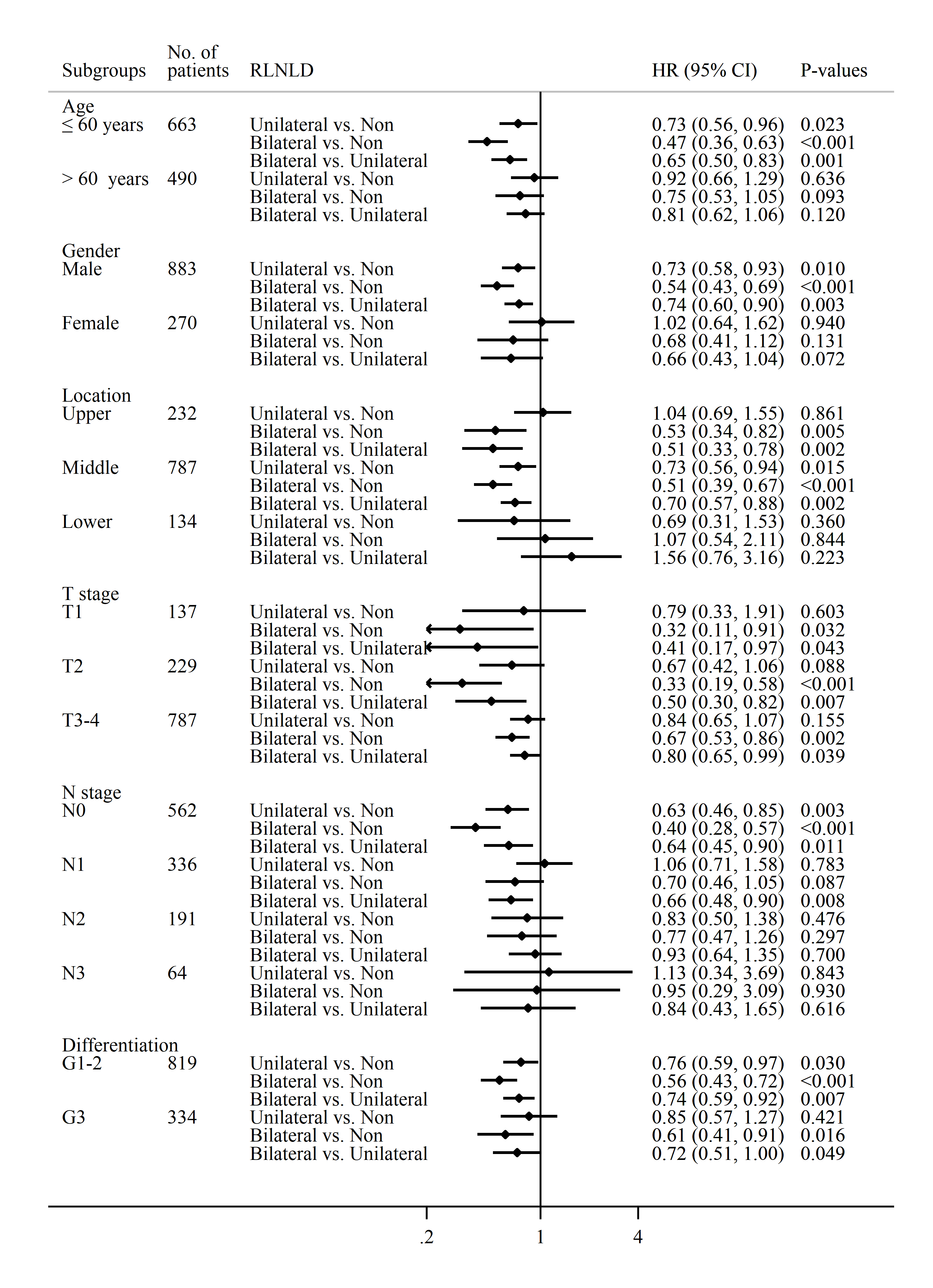

Supplement: Supplementary file 2 — Fig S2 [file CAM4-11-1617-s001.tif]

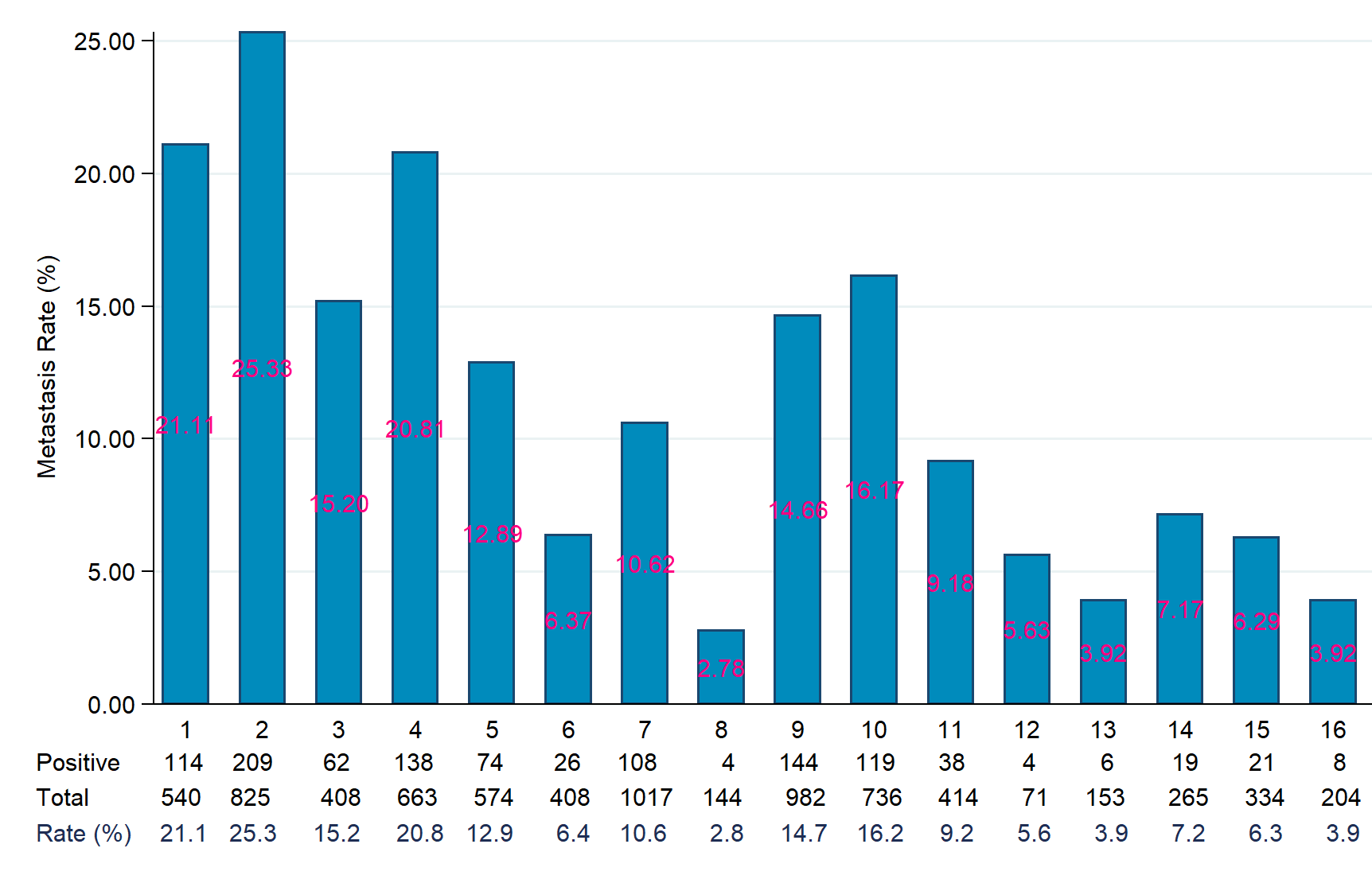

Supplement: Supplementary file 3 — Fig S3 [file CAM4-11-1617-s002.tif]

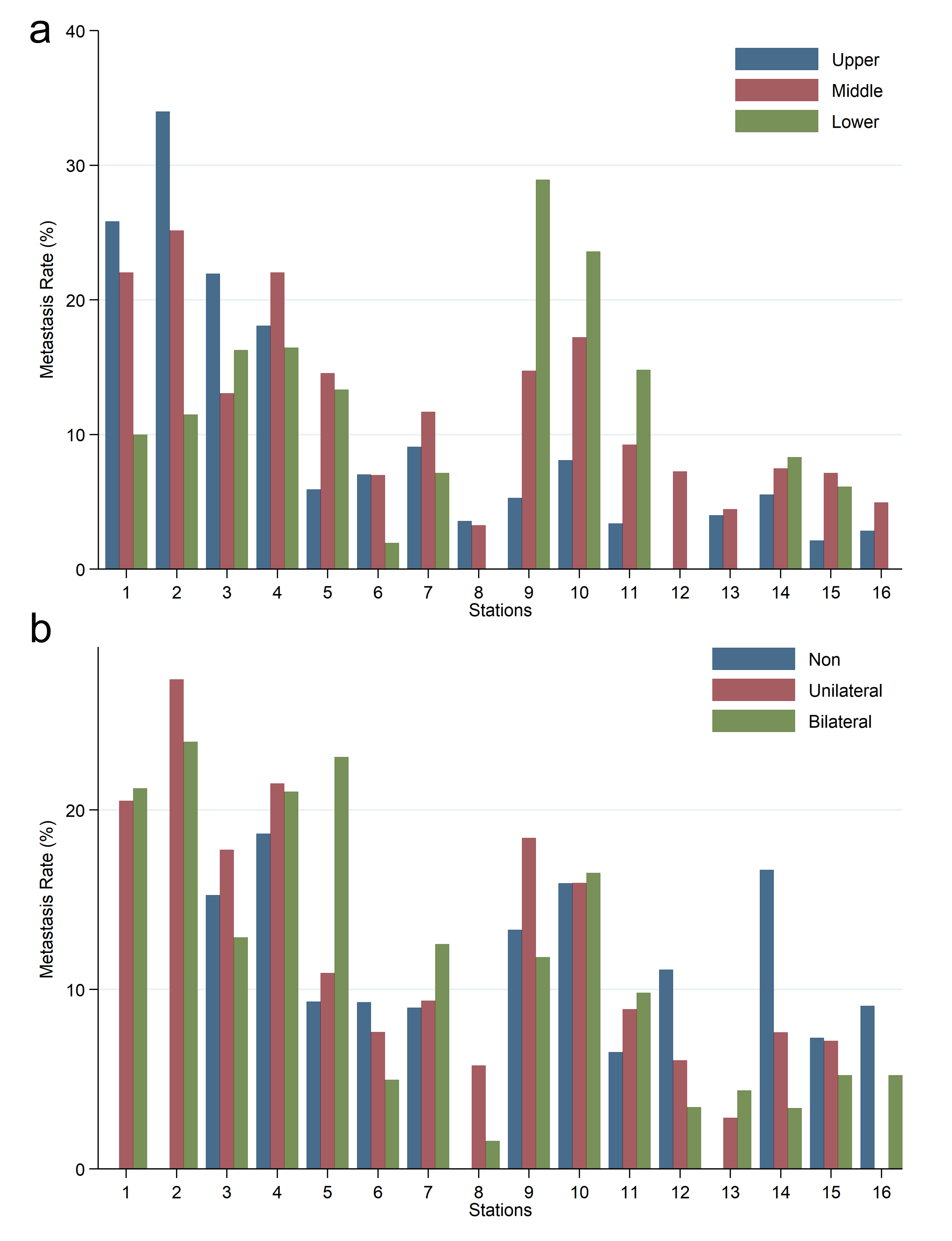

Supplement: Supplementary file 4 — Fig S4 [file CAM4-11-1617-s005.tif]
