# Supplementary material for: Clinical significance and outcomes of bilateral and unilateral recurrent laryngeal nerve lymph node dissection in esophageal squamous cell carcinoma: A large‐scale retrospective cohort study
Source: Cancer Med. 2022 Feb 17;11(7):1617–29. doi: 10.1002/cam4.4399 (PMC8986140; doi:10.1002/cam4.4399)
Supplement: Supplementary file 5 — Table S1‐S3 [file CAM4-11-1617-s004.doc]

Supplement Table 1. The clinical and pathologic characteristics at baseline

| Characteristic | Before IPTW | | |  | After IPTW | | |
| --- | --- | --- | --- | --- | --- | --- | --- |
|  | Non RLNLD (n=250) | Bilateral RLNLD (n=462) | P-value |  | Non RLNLD (n=720.9) | Bilateral RLNLD (n=709.1) | P-value |
| Age |  |  | 0.324 |  |  |  | 0.948 |
| ≤ 60 years | 154 (61.6) | 267 (57.8) |  |  | 423.6 (58.8) | 418.6 (59.0) |  |
| > 60 years | 96 (38.4) | 195 (42.2) |  |  | 297.2 (41.2) | 290.5 (41.0) |  |
| Gender |  |  | **0.005** |  |  |  | 0.808 |
| Females | 72 (28.8) | 90 (19.5) |  |  | 153.9 (21.3) | 156.9 (22.1) |  |
| Males | 178 (71.2) | 372 (80.5) |  |  | 567.0 (78.7) | 552.2 (77.9) |  |
| Smoking |  |  | 0.181 |  |  |  | 0.940 |
| Never | 102 (40.8) | 165 (35.7) |  |  | 266.4 (37.0) | 264.2 (37.3) |  |
| Ever (former + current) | 148 (59.2) | 297 (64.3) |  |  | 454.5 (63.0) | 444.9 (62.7) |  |
| Alcohol |  |  | 0.151 |  |  |  | 1.000 |
| Never | 173 (69.2) | 295 (63.9) |  |  | 471.9 (65.5) | 464.2 (65.5) |  |
| Ever (former + current) | 77 (30.8) | 167 (36.1) |  |  | 249.0 (34.5) | 244.9 (34.5) |  |
| pT stage |  |  | 0.419 |  |  |  | 0.899 |
| T1-2 | 82 (32.8) | 138 (29.9) |  |  | 218.4 (30.3) | 218.3 (30.8) |  |
| T3-4 | 168 (67.2) | 324 (70.1) |  |  | 502.4 (69.7) | 490.8 (69.2) |  |
| pN stage |  |  | **< 0.001** |  |  |  | 0.884 |
| N0 | 159 (63.6) | 193 (41.8) |  |  | 349.8 (48.5) | 348.4 (49.1) |  |
| N1-3 | 91 (36.4) | 269 (58.2) |  |  | 371.0 (51.5) | 360.6 (50.9) |  |
| Differentiation |  |  | 0.323 |  |  |  | 0.818 |
| G1-2 | 182 (72.8) | 320 (69.3) |  |  | 500.5 (69.4) | 498.7 (70.3) |  |
| G3 | 68 (27.2) | 142 (30.7) |  |  | 220.4 (30.6) | 210.3 (29.7) |  |
| Tumor location |  |  | **0.001** |  |  |  | 0.980 |
| Upper | 70 (28.0) | 77 (16.7) |  |  | 140.1 (19.4) | 142.3 (20.1) |  |
| Middle | 148 (59.2) | 330 (71.4) |  |  | 492.3 (68.3) | 479.8 (67.7) |  |
| Lower | 32 (12.8) | 55 (11.9) |  |  | 88.5 (12.3) | 87.0 (12.3) |  |
| Adjuvant therapy |  |  | 0.224 |  |  |  | 0.760 |
| No | 208 (83.2) | 367 (79.4) |  |  | 573.5 (79.6) | 571.6 (80.6) |  |
| Yes | 42 (16.8) | 95 (20.6) |  |  | 147.4 (20.4) | 137.4 (19.4) |  |
| Postoperative Complication |  |  | **0.004** |  |  |  | 0.908 |
| No | 163 (65.2) | 250 (54.1) |  |  | 414.2 (57.5) | 410.9 (57.9) |  |
| Yes | 87 (34.8) | 212 (45.9) |  |  | 306.7 (42.5) | 298.2 (42.1) |  |

Supplement Table 2. The clinical and pathologic characteristics at baseline

| Characteristic | Before IPTW | | |  | After IPTW | | |
| --- | --- | --- | --- | --- | --- | --- | --- |
|  | Non RLNLD (n=250) | Unilateral RLNLD (n=441) | P-value |  | Non RLNLD (n= 692.0) | Unilateral RLNLD (n= 690.9) | P-value |
| Age |  |  | 0.086 |  |  |  | 0.872 |
| ≤ 60 years | 154 (61.6) | 242 (54.9) |  |  | 404.0 (58.4) | 398.8 (57.7) |  |
| > 60 years | 96 (38.4) | 199 (45.1) |  |  | 288.0 (41.6) | 292.1 (42.3) |  |
| Gender |  |  | 0.215 |  |  |  | 0.930 |
| Females | 72 (28.8) | 108 (24.5) |  |  | 177.1 (25.6) | 178.9 (25.9) |  |
| Males | 178 (71.2) | 333 (75.5) |  |  | 514.9 (74.4) | 512.0 (74.1) |  |
| Smoking |  |  | 0.685 |  |  |  | 0.880 |
| Never | 102 (40.8) | 173 (39.2) |  |  | 280.5 (40.5) | 275.8 (39.9) |  |
| Ever (former + current) | 148 (59.2) | 268 (60.8) |  |  | 411.5 (59.5) | 415.0 (60.1) |  |
| Alcohol |  |  | 0.942 |  |  |  | 0.898 |
| Never | 173 (69.2) | 304 (68.9) |  |  | 482.2 (69.7) | 478.1 (69.2) |  |
| Ever (former + current) | 77 (30.8) | 137 (31.1) |  |  | 209.8 (30.3) | 212.8 (30.8) |  |
| pT stage |  |  | 0.934 |  |  |  | 0.924 |
| T1-2 | 82 (32.8) | 146 (33.1) |  |  | 231.7 (33.5) | 228.8 (33.1) |  |
| T3-4 | 168 (67.2) | 295 (66.9) |  |  | 460.3 (66.5) | 462.1 (66.9) |  |
| pN stage |  |  | **< 0.001** |  |  |  | 0.996 |
| N0 | 159 (63.6) | 210 (47.6) |  |  | 369.6 (53.4) | 369.2 (53.4) |  |
| N1-3 | 91 (36.4) | 231 (52.4) |  |  | 322.4 (46.6) | 321.7 (46.6) |  |
| Differentiation |  |  | 0.796 |  |  |  | 0.913 |
| G1-2 | 182 (72.8) | 317 (71.9) |  |  | 495.6 (71.6) | 497.6 (72.0) |  |
| G3 | 68 (27.2) | 124 (28.1) |  |  | 196.4 (28.4) | 193.3 (28.0) |  |
| Tumor location |  |  | **0.012** |  |  |  | 0.988 |
| Upper | 70 (28.0) | 85 (19.3) |  |  | 150.1 (21.7) | 153.1 (22.2) |  |
| Middle | 148 (59.2) | 309 (70.1) |  |  | 462.9 (66.9) | 458.3 (66.3) |  |
| Lower | 32 (12.8) | 47 (10.7) |  |  | 79.0 (11.4) | 79.4 (11.5) |  |
| Adjuvant therapy |  |  | 0.246 |  |  |  | 0.929 |
| No | 208 (83.2) | 351 (79.6) |  |  | 558.5 (80.7) | 559.6 (81.0) |  |
| Yes | 42 (16.8) | 90 (20.4) |  |  | 131.3 (19.0) | 133.5 (19.3) |  |
| Postoperative Complication |  |  | 0.974 |  |  |  | 0.977 |
| No | 163 (65.2) | 287 (65.1) |  |  | 449.8 (65.0) | 449.8 (65.1) |  |
| Yes | 87 (34.8) | 154 (34.9) |  |  | 242.2 (35.0) | 241.1 (34.9) |  |

Supplement Table 3. The clinical and pathologic characteristics at baseline

| Characteristic | Before IPTW | | |  | After IPTW | | |
| --- | --- | --- | --- | --- | --- | --- | --- |
|  | Unilateral RLNLD (n=441) | Bilateral RLNLD (n=462) | P-value |  | Unilateral RLNLD (n=903.6) | Bilateral RLNLD (n=902.4) | P-value |
| Age |  |  | 0.377 |  |  |  | 0.994 |
| ≤ 60 years | 242 (54.9) | 267 (57.8) |  |  | 505.3 (55.9) | 504.9 (55.9) |  |
| > 60 years | 199 (45.1) | 195 (42.2) |  |  | 398.2 (44.1) | 397.5 (44.1) |  |
| Gender |  |  | 0.069 |  |  |  | 0.994 |
| Females | 108 (24.5) | 90 (19.5) |  |  | 196.5 (21.7) | 196.0 (21.7) |  |
| Males | 333 (75.5) | 372 (80.5) |  |  | 707.1 (78.3) | 706.4 (78.3) |  |
| Smoking |  |  | 0.275 |  |  |  | 0.984 |
| Never | 173 (39.2) | 165 (35.7) |  |  | 339.9 (37.6) | 338.8 (37.5) |  |
| Ever (former + current) | 268 (60.8) | 297 (64.3) |  |  | 563.7 (62.4) | 563.6 (62.5) |  |
| Alcohol |  |  | 0.106 |  |  |  | 0.986 |
| Never | 304 (68.9) | 295 (63.9) |  |  | 597.6 (66.1) | 597.4 (66.2) |  |
| Ever (former + current) | 137 (31.1) | 167 (36.1) |  |  | 306.0 (33.9) | 305.1 (33.8) |  |
| pT stage |  |  | 0.295 |  |  |  | 0.980 |
| T1-2 | 146 (33.1) | 138 (29.9) |  |  | 282.8 (31.3) | 281.7 (31.2) |  |
| T3-4 | 295 (66.9) | 324 (70.1) |  |  | 620.8 (68.7) | 620.7 (68.8) |  |
| pN stage |  |  | 0.077 |  |  |  | 0.991 |
| N0 | 210 (47.6) | 193 (41.8) |  |  | 403.8 (44.7) | 403.0 (44.7) |  |
| N1-3 | 231 (52.4) | 269 (58.2) |  |  | 499.7 (55.3) | 499.4 (55.3) |  |
| Differentiation |  |  | 0.388 |  |  |  | 0.967 |
| G1-2 | 317 (71.9) | 320 (69.3) |  |  | 635.1 (70.3) | 635.4 (70.4) |  |
| G3 | 124 (28.1) | 142 (30.7) |  |  | 268.5 (29.7) | 267.0 (29.6) |  |
| Tumor location |  |  | 0.542 |  |  |  | 0.999 |
| Upper | 85 (19.3) | 77 (16.7) |  |  | 162.2 (18.0) | 161.7 (17.9) |  |
| Middle | 309 (70.1) | 330 (71.4) |  |  | 641.4 (71.0) | 640.0 (70.9) |  |
| Lower | 47 (10.7) | 55 (11.9) |  |  | 99.9 (11.1) | 100.7 (11.2) |  |
| Adjuvant therapy |  |  | 0.954 |  |  |  | 0.995 |
| No | 351 (79.6) | 367 (79.4) |  |  | 721.3 (79.8) | 720.2 (79.8) |  |
| Yes | 90 (20.4) | 95 (20.6) |  |  | 182.3 (20.2) | 182.2 (20.2) |  |
| Postoperative Complication |  |  | **0.001** |  |  |  | 1.000 |
| No | 287 (65.1) | 250 (54.1) |  |  | 535.8 (59.3) | 535.1 (59.3) |  |
| Yes | 154 (34.9) | 212 (45.9) |  |  | 367.7 (40.7) | 367.3 (40.7) |  |
